# Early Changes in the Locus Coeruleus in Mild Cognitive Impairment with Lewy Bodies

**DOI:** 10.1002/mds.30058

**Published:** 2024-11-13

**Authors:** Žaneta Železníková, L'ubomíra Nováková, Lubomír Vojtíšek, Luboš Brabenec, Kristína Mitterová, Ivona Morávková, Irena Rektorová

**Affiliations:** ^1^ Applied Neuroscience Research Group, Central European Institute of Technology, CEITEC Masaryk University Brno Czech Republic; ^2^ International Clinical Research Center, ICRC Faculty of Medicine and St. Anne's University Hospital, Masaryk University Brno Czech Republic; ^3^ Multimodal and Functional Neuroimaging Research Group, Central European Institute of Technology, CEITEC Masaryk University Brno Czech Republic; ^4^ First Department of Neurology Faculty of Medicine and St. Anne's University Hospital, Masaryk University Brno Czech Republic

**Keywords:** mild cognitive impairment with Lewy bodies, neuromelanin‐sensitive magnetic resonance imaging, locus coeruleus

## Abstract

**Background:**

Although neuromelanin‐sensitive magnetic resonance imaging (NM‐MRI) has been used to evaluate early neurodegeneration in Parkinson's disease, studies concentrating on the locus coeruleus (LC) in pre‐dementia stages of dementia with Lewy bodies (DLB) are lacking.

**Objectives:**

The aims were to evaluate NM‐MRI signal changes in the LC in patients with mild cognitive impairment with Lewy bodies (MCI‐LB) compared to healthy controls (HC) and to identify the cognitive correlates of the changes. We also aimed to test the hypothesis of a caudal‐rostral α‐synuclein pathology spread using NM‐MRI of the different LC subparts.

**Methods:**

A total of 38 MCI‐LB patients and 59 HCs underwent clinical and cognitive testing and NM‐MRI of the LC. We calculated the contrast ratio of NM‐MRI signal (LC‐CR) in the whole LC as well as in its caudal, middle, and rostral MRI slices, and we compared the LC‐CR values between the MCI‐LB and HC groups. Linear regression analyses were performed to assess the relationship between the LC‐CR and cognitive outcomes.

**Results:**

The MCI‐LB group exhibited a significant reduction in the right LC‐CR compared to HCs (*P* = 0.021). The right LC‐CR decrease was associated with impaired visuospatial memory in the MCI‐LB group. Only the caudal part of the LC exhibited significant LC‐CR decreases in MCI‐LB patients compared to HCs on both sides (*P* < 0.0001).

**Conclusions:**

This is the first study that focuses on LC‐CRs in MCI‐LB patients and analyzes the LC subparts, offering new insights into the LC integrity alterations in the initial stages of DLB and their clinical correlates. © 2024 The Author(s). *Movement Disorders* published by Wiley Periodicals LLC on behalf of International Parkinson and Movement Disorder Society.

A new classification of Parkinson's disease (PD) with the SynNeurGe research diagnostic criteria was recently published, suggesting the documentation of pathological α‐synuclein in tissues or cerebrospinal fluid and/or pathogenic gene variants that induce or cause a strong predisposition to PD.^1^ These results provide only “yes” or “no” answers; the evaluation of neurodegeneration using neuroimaging tools has also been recommended to better characterize the spatial distribution, temporal evolution, and magnitude of the changes. Another paper^2^ directly referred to neuronal α‐synuclein diseases. The authors provided a staging system for research based on the detection of pathological α‐synuclein, dopaminergic neuronal dysfunction, the presence of specific clinical symptoms, and functional impairment. Although the two aforementioned papers presented important breakthroughs for diagnosing Lewy body diseases in research, a mechanistic understanding of how the temporal spread of α‐synuclein pathology in the brain affects motor symptoms and cognitive and other nonmotor symptoms of the disease is also warranted for developing disease‐modifying treatment. In this regard, brain alteration patterns, as assessed using noninvasive and widely available MRI, may provide important insights into the development of distinct clinical phenotypes, their temporal evolution, and disease prognosis.^3^, ^4^, ^5^

An interesting hypothesis has been formulated concerning the temporal spread of α‐synuclein pathology^6^ originating either in the peripheral nervous system and gastrointestinal tract in particular, spreading in a prion‐like manner toward the brain following Braak staging (“body‐first” subtype), or starting asymmetrically in the amygdala and limbic system and from there following a rostral‐caudal pattern of Lewy body pathology and spreading to the peripheral system (“brain‐first” subtype). In line with this, the authors suggested the presence of early cognitive decline and an overall malignant course of the disease in the former (dementia with Lewy bodies [DLB]) subtype and an earlier more pronounced asymmetric parkinsonism (typical for less‐malignant PD phenotype) in the latter subtype.^7^, ^8^, ^9^

Probably the best‐described phenotype of pre‐dementia DLB is mild cognitive impairment with Lewy bodies (MCI‐LB), which can be distinguished from other causes of MCI by the presence of core clinical features of DLB and diagnostic biomarkers such as reduced dopamine transporter uptake in the basal ganglia as assessed using dopaminergic single‐photon emission computed tomography/positron emission tomography (SPECT/PET), reduced cardiac metaiodobenzylguanidine uptake on myocardial scintigraphy, and polysomnography‐diagnosed rapid eye movement (REM) sleep without atonia.^10^ Other supportive features and biomarkers have also been documented.^10^, ^11^, ^12^, ^13^

However, a recent meta‐analysis revealed that sensitivity of FP‐CIT SPECT (2β‐carbomethoxy)‐3β‐(4‐iodophenyl)‐*N*‐(3‐fluoropropyl) nortropane was rather low for diagnosing MCI‐LB (65%–66%).^11^ This could be because in early MCI‐LB, the α‐synuclein pathology affects the locus coeruleus (LC) prior to the substantia nigra pars compacta (SNpc, in accordance with the aforementioned “body‐first” hypothesis), which is consistent with a typical cognitive profile of MCI‐LB, that is, executive and attention deficits in particular.^14^

To test this hypothesis, we used neuromelanin‐sensitive magnetic resonance imaging (NM‐MRI) of the LC and its caudal, intermediate, and rostral subparts. The LC produces norepinephrine, a neurotransmitter that regulates arousal, attention, autonomic functions, and the sleep–wake cycle, and that also plays a crucial role in stress response and depression, emotional and episodic memory, and working memory and executive functions.^15^, ^16^ Neuromelanin (NM), as a by‐product of the catecholamine metabolism, is a biochemical‐insoluble pigment of dopaminergic neurons in the SNpc and in the noradrenergic neurons in the LC. The NM bound to the metal is paramagnetic, and paramagnetic substances shorten the T1 relaxation time.^17^ This allows the visualization of structures containing NM, such as the SNpc and the LC, as high‐intensity areas; the signal from the surrounding brain tissue is suppressed. NM levels are known to decline with neurodegeneration,^18^ and NM‐MRI of the SNpc has been particularly useful for evaluating SNpc neurodegeneration early in the course of PD,^19^, ^20^ and in patients with REM sleep behavioral disorder (RBD).^21^, ^22^ Moreover, NM‐MRI seems suitable for monitoring disease progression.^23^, ^24^

Although NM‐MRI of the LC has been used in PD,^16^, ^25^, ^26^ to the best of our knowledge no study has yet used NM‐MRI to study LC changes in clinically well‐defined MCI‐LB patients. We tested the utility of NM‐MRI as a potential marker of the early neurodegeneration of the LC in MCI‐LB compared to healthy controls (HC). We specifically concentrated on studying the LC for two reasons: (1) pathological changes affect this area early according to the body‐first hypothesis; (2) LC disruption is related to cognitive, autonomic, and psychiatric functions,^15^, ^27^ which are predominant in DLB patients, whereas motor symptoms dominate in early PD and are rather closely related to SNpc disruption.^28^ We hypothesized a measurable reduction in LC signal in MCI‐LB compared to age‐matched HCs. In line with the body‐first hypothesis, we predicted that the differences between MCI‐LB and HC are more pronounced in the caudal subparts of the LC. We also hypothesized that in the MCI‐LB group, the MRI signal in the LC correlates with specific MCI‐LB cognitive deficits.

In a majority of DLB patients, Alzheimer's disease (AD) brain pathology may coexist with α‐synuclein pathology and may influence its clinical manifestation.^29^ AD‐related pathology has been described in the LC of AD patients.^30^ To evaluate whether any significant correlations between the LC MRI signal and cognitive outcomes are modified by AD‐related co‐pathology in our MCI‐LB cohort, we used a validated blood‐based immunoassay measuring plasma pTau181 as a biomarker of AD‐related brain pathology^31^, ^32^, ^33^ in individual subjects.

## Patients and Methods

### Participants

This study included 38 participants who met the criteria for MCI‐LB.^10^ We included 59 participants as HCs. For recruitment procedures and inclusion and exclusion criteria, see Supplementary Material. During the in‐person screening visit, we collected a detailed medical history and conducted a neurological and cognitive examination; for details, see Supplementary Material.

All participants were informed in detail about the study and signed an informed consent form approved by the local ethics committee. All data were handled in line with the General Data Protection Regulation (GDPR). Based on research criteria for diagnosis of MCI‐LB,^10^ a cognitive complaint was required from the patient or from an informant or clinician, and a cognitive profile of MCI‐LB was evaluated by MDS (the International Parkinson and Movement Disorder Society) level II criteria for PD‐MCI.^34^ We included MCI subjects with two neuropsychological tests with a cutoff score under −1 standard deviation (SD) below the age‐appropriate norms or with one test with a cutoff score under −1 SD below age‐appropriate norms plus a Montreal Cognitive Assessment cutoff score of 26 with the coexistence of at least two core clinical features of DLB according to the results of a detailed clinical examination, patient interview, and cutoff scores of validated scales/questionnaires evaluating fluctuating cognition, visual hallucinations, RBD, and one or more spontaneous cardinal features of parkinsonism^10^, ^35^ (see Supplementary Material for details). Based on the clinician's interview with a patient and a caregiver, the cognitive deficits did not interfere with patients' daily functioning. HCs did not have MCI or meet any core clinical symptoms of MCI‐LB. All of our participants underwent plasma sample collection through venipuncture. Plasma pTau181 was evaluated by our partner institution (Clinical Neurochemistry Laboratory, University of Gothenburg, Sweden) in a blinded manner, on the Simoa HD‐1 (Quanterix, Billerica, MA, USA) as described previously.^32^

### Image Acquisition

All magnetic resonance images were acquired using the 3 T Siemens Magnetom Prisma. A three‐dimensional brain volume imaging sequence was performed for the whole brain T1 mprage structural data with the following parameters: repetition time (TR) = 2400.0 ms, echo time (TE) = 2.27 ms, inversion time (TI) = 1150 ms, field of view (FOV) = 218 × 218 mm, matrix size = 256 × 256, flip angle = 8°, bandwidth = 210 Hz/Px, averages = 1, in‐plane resolution = 0.85 × 0.85 mm, slice thickness = 0.85 mm with no intersection gap, and number of slices = 192. The LC NM‐MRI scans were performed using a T1‐weighted fast spin‐echo sequence with the following parameters: TR = 890.0 ms, TE = 13 ms, in‐plane resolution = 0.4 × 0.4 mm, slice thickness = 3.0 mm, matrix size = 512 × 464, nine slices, FOV = 220 × 200 mm, flip angle = 180°, Magnetization Transfer Contrast (MTC) on, bandwidth = 160 Hz/Px, and number of averages = 3.

### Image Processing and Accessing the Neuromelanin Signal

To process the data, we employed a combination of software tools, including Advanced Normalization Tools (ANT, version 2.2.0), Matlab (MATLAB R2017b), and the FMRIB Software Library. Initially, T1‐weighted images were skull stripped using ANTs' antsBrainExtraction.sh. This was followed by spatial normalization to the MNI152NLinAsym template space using antsRegistrationSyN.sh (incorporating rigid, affine, and deformable syn transformations). Subsequently, we coregistered the NM‐MRI image to the T1w (in native space) image using antsRegistrationSyN.sh with rigid transformations. Finally, we executed antsApplyTransforms to integrate the previously derived transformations, facilitating a one‐step normalization of the LC and pontine tegmentum (PT) masks to the NM‐MRI image.

**FIG. 1:**
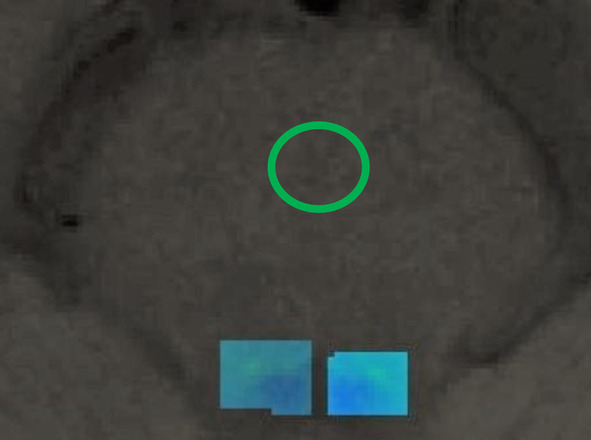
The manually drawn overinclusive mask (two rectangular shapes) projected on a sample participant's neuromelanin (NM) image; the circle is a reference region of the pontine tegmentum. [Color figure can be viewed at wileyonlinelibrary.com]

To access the LC signals, we adopted a methodology comparable to one used in previous research.^36^, ^37^, ^38^ A manually delineated overinclusive mask served as the search area for the LC (see Fig. 1). For the reference region, we drew the region of interest (ROI) in the central PT (see Fig. 1). This reference area was selected based on past studies due to its high signal‐to‐noise ratio. It is thought to remain unaffected by any pathogenesis, making it appropriate for intensity normalization to counteract variances between subjects and slices.^39^ Within each slice, the intensity was linearly modified so that the mean intensity of the reference region was uniform across all images and set to the same predefined value. For further analyses, we used the contrast ratio (CR) between the LC and the central PT using formula (1):

(1) $$\mathrm{LC}-\mathrm{CR}=({\mathrm{LC}}_{\text{intensity}}-{\mathrm{PT}}_{\text{intensity}})/{\mathrm{PT}}_{\text{intensity}}$$

In these overinclusive masks on both the right and left sides, we localized the voxel with the highest intensity that aligned with the anatomical position of the LC. A cross of five voxels was then placed at this peak voxel to cover the LC's neuronal distribution of 1 to 2 mm. If the peak voxel was adjacent to the fourth ventricle, we shifted the center voxel one step away to avoid underestimating the signal intensity. This ensured the peak voxel stayed within the LC cross ROI. The signal was then measured in three slices denoting the rostral, middle, and caudal LC (see Supplementary Material), with the middle slice positioned 7 mm below the inferior edge of the inferior colliculus, in line with the existing literature.^37^, ^40^

### Statistical Methods

All statistical analyses were performed using software from MATLAB, Python, and SPSS, and R. Demographic, clinical data, and LC‐CR (controlling for the effect of age) were expressed as the mean and SD and were analyzed using the 1‐tailed Mann‐Whitney *U* test. The χ^2^ test was used to compare categorical variables such as sex. To investigate the association between LC‐CR and cognitive performance (see Table 1), linear regression analyses were used, controlling for the effect of age and education and adhering to the assumptions of linear regression (see Supplementary Material). In addition, using Spearman's correlations we exploratively assessed the correlation between LC‐CR and other nonmotor scores that might have revealed significant associations based on the literature,^16^ such as the Geriatric Depression Scale (GDS), Epworth Sleepiness Scale (ESS), and REM Sleep Behavior Disorder Screening Questionnaire (RBDSQ). Finally, to assess whether any significant associations between LC‐CR and cognitive outcomes of interest were modified by AD brain co‐pathology, we additionally regressed out the effect of plasma pTau181 levels.^32^

**TABLE 1:** Demographic, clinical, and cognitive data

	HC mean ± SD	MCI‐LB mean ± SD	*U*	*P*
Sex (female:male)	20:18	39:20		0.185
Age	65.72 ± 7.14	69.65 ± 5.86	1514	0.004
Education (y)	16.14 ± 0.45	13.83 ± 0.58	651	<0.001
MoCA	27.38 ± 1.39	23.92 ± 3.06	324	<0.001
Visuospatial functions *z* score	0.17 ± 0.77	−0.50 ± 1.12	724	0.004
Memory *z* score	0.58 ± 0.64	−0.37 ± 0.83	387.5	<0.001
Attention *z* score	0.26 ± 0.59	−0.23 ± 0.68	652.5	<0.001
Executive functions *z* score	0.32 ± 0.75	−0.41 ± 0.84	602	<0.001
GDS score	6.88 ± 5.11	8.21 ± 6.26	1220	0.376
RBDSQ score	2.86 ± 2.16	4.16 ± 2.59	1420.5	0.016
ESS score	7.50 ± 4.34	8.03 ± 3.98	1223	0.363
UPDRS score	2.58 ± 3.48	6.37 ± 4.39	1750	<0.001

We used the Mann‐Whitney *U* test for age and the *χ* ^2^ test for sex comparison between the MCI‐LB and HC groups.

Abbreviations: HC, healthy control; SD, standard deviation; MCI‐LB, mild cognitive impairment with Lewy bodies; MoCA, Montreal Cognitive Assessment; GDS, Geriatric Depression Scale; RBDSQ, Rapid Eye Movement Sleep Behavior Disorder Screening Questionnaire; ESS, Epworth Sleepiness Scale; UPDRS, Unified Parkinson's Disease Rating Scale.

## Results

There were no significant sex differences between the MCI‐LB patients and the HC group, but there was a significant difference in age and education. Table 1 presents the demographic, clinical, and cognitive data for both groups.

### Neuromelanin‐Sensitive magnetic resonance imaging

**FIG. 2:**
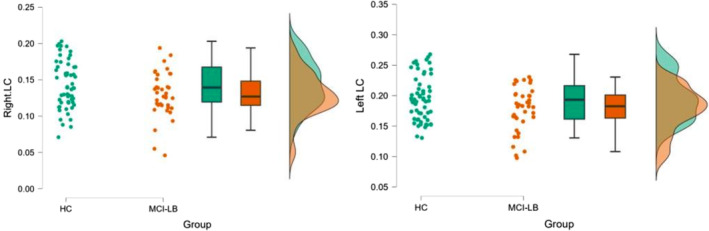
Raincloud plots of the differences between both groups for both the left and the right LC‐CR (locus coeruleus‐contrast ratio). [Color figure can be viewed at wileyonlinelibrary.com]

**FIG. 3:**
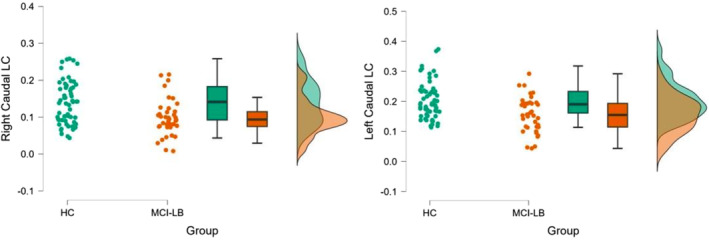
Raincloud plots showing the difference between mild cognitive impairment with Lewy bodies (MCI‐LB) and healthy controls (HC) for the right‐ and the left‐caudal LC‐CR (locus coeruleus‐contrast ratio). [Color figure can be viewed at wileyonlinelibrary.com]

We observed a significantly reduced signal in the right LC of the MCI‐LB group compared to the HC group (*P* = 0.021); there was a similar nonsignificant trend on the left side (*P* = 0.055); see Figure 2. When the LC subparts were analyzed in separate slices (see Patients and Methods), we found a significant signal reduction on both sides only for the caudal part of the LC in MCI‐LB compared to the HC group (*P* < 0.0001); see Figure 3. Additional results are presented in detail in the Supplementary Material.

### Linear Regressions

**FIG. 4:**
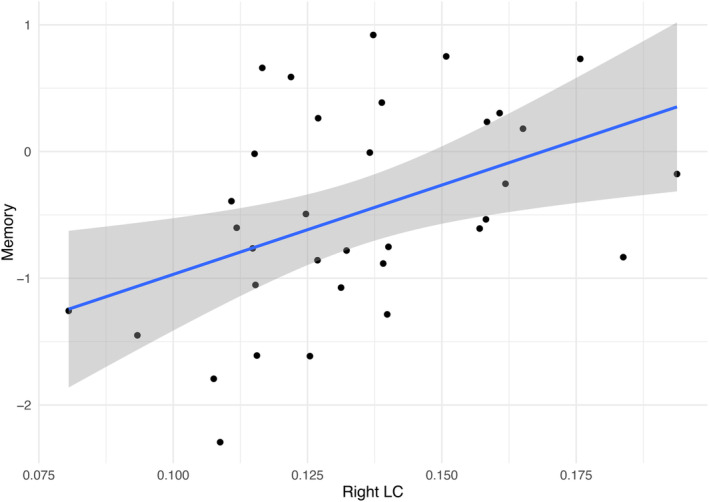
Scatterplot of significant association between the right LC‐CR (locus coeruleus‐contrast ratio) and the memory *z* score of the mild cognitive impairment with Lewy bodies (MCI‐LB) group. [Color figure can be viewed at wileyonlinelibrary.com]

Linear regression analyses, controlling for age and education, were conducted to examine the associations between LC‐CR and various cognitive domains. The right LC‐CR significantly predicted the memory domain (F(3, 33) = 3.49, *ß* = 14.72, *P* = 0.01), as shown in Figure 4 and the Supplementary Material, which includes regression parameters and diagnostics. However, the right LC‐CR only marginally predicted visuospatial functions (F(3, 36) = 3.38, *ß* = 11.46, *P* = 0.053). Additionally, the left LC‐CR did not significantly predict either the memory or visuospatial domains.

Further inspection of the memory domain revealed that the right LC‐CR specifically predicted the sum of immediate recall trials, as measured by the Brief Visuospatial Memory test (BVMT)_2 *z* score (F(4, 33) = 2.88, *ß* = 19.38, *P* = 0.011); this relationship remained significant even when accounting for the levels of plasma pTau181 (*P* = 0.514; see Supplementary Material). Other scales within the memory domain were not significantly predicted.

Exploratory analyses of LC subparts indicated a positive association between visuospatial function *z* scores and both the right‐middle LC (F(1, 36) = 4.77, *ß* = 8.31, *P* = 0.036) and the left‐middle LC (F(1, 36) = 4.21, *ß* = 6.52, *P* = 0.048). Furthermore, the right‐middle LC significantly predicted the memory *z* score (F(1, 33) = 7.96, *ß* = 9.44, *P* = 0.008).

### Correlation Analyses

Regarding the exploratory analyses of other nonmotor scores (GDS, ESS, RBDSQ), we found a significant negative correlation between the left LC‐CR and GDS (rho = − 0.303, *P* = 0.032); see figure in Supplementary Material. Exploratory correlations with the LC subparts exhibited a negative correlation between the right caudal LC and RBDSQ (rho = −0.309, *P* = 0.029) and between the right‐middle LC and GDS (rho = −0.378, *P* = 0.01) and the left‐middle LC and GDS (rho = −0.357, *P* = 0.014).

## Discussion

This is the first study that focuses on LC integrity and its clinical correlates specifically in MCI‐LB patients compared to HCs, including an evaluation of distinct LC subparts using NM‐MRI. Moreover, plasma pTau181 values were implemented in data analysis to provide information on the influence of AD‐related co‐pathology on associations between LC‐CR and cognitive outcomes of interest in the MCI‐LB cohort.

We observed decreased integrity of the right LC in the MCI‐LB group compared to the HC group with a similar nonsignificant trend on the left side. When we zoomed in on the LC‐CR in separate slices, we found only the caudal part of the left and right LC significantly affected in MCI‐LB compared to HCs. Moreover, the exploratory analysis showed that the caudal part of the right LC was negatively correlated with the RBDSQ scores, meaning that the more pronounced the degeneration of the caudal LC, the more pronounced the symptoms of RBD. Although we found no studies investigating NM in different parts of the LC in DLB, similar findings of caudal LC alterations were mentioned in previous studies of PD,^25^, ^26^, ^41^, ^42^ which reported that the decrease in LC‐CR was not evenly distributed throughout the LC and that the middle and caudal parts of the LC were more affected than the rostral part. In line with our study results, Nobileau and colleagues^43^ demonstrated that LC signal intensity was reduced in PD with RBD but not in PD without RBD. In the same vein, the disruption of LC‐related functional networks was seen in PD with RBD; these networks were less affected in PD without RBD.^44^ Overall, our result of the caudal LC degeneration in MCI‐LB further supports the “body‐first” hypothesis in this more malignant neuronal α‐synuclein disease population^8^, ^45^ and underscores the potential of NM‐MRI as a biomarker for identifying candidates for specific strategies of disease modification and prevention.^9^

Specific disruptions of LC segments may be disease specific; for example, in the early stages of AD, the rostral portions of LC are the most affected.^46^ In the current work, the right LC‐CR exhibited significant association with memory functions, especially with the sum of BVMT immediate recall trials, which is a measure of visual and spatial short‐term memory and the learning abilities of the subject.^47^ Of note, the exploratory analyses revealed that the visuospatial functions and memory LC‐CR associations were particularly with the middle subpart of the LC, which is in accordance with the functional organization of the LC.^15^ Visual memory and visuospatial ability impairments are characteristic of MCI‐LB,^48^ and visual memory performance in DLB is poorer than in Parkinson's disease dementia^49^ and equally poor^50^, ^51^ or even worse than in AD.^52^

The current results are in line with the suggestion that noradrenergic neurons in the LC play a critical role in learning and memory,^53^, ^54^ due to its well‐described neural projections to the amygdala, hippocampus, and prefrontal cortex.^15^, ^55^ Because AD‐related brain pathology may co‐occur in DLB^29^ and may also be associated with visuospatial memory impairment,^56^ we assessed the impact of pTau181 plasma levels, that is, a biomarker of AD brain pathology,^32^ on the association between LC‐CR and BVMT. We did not observe a major impact of the AD plasma biomarker levels on the relation, suggesting that the association of the NM‐MRI results with cognitive outcomes of interest remains rather specific for the neuronal α‐synuclein disease.

We also conducted an exploratory analysis focused on depressive symptoms. The results showed a significant negative correlation between the LC‐CR and GDS score in the MCI‐LB group; depressive symptoms tended to increase with LC neurodegeneration. The LC is known to play a key role in mood regulation, and its degeneration has been implicated in various mood disorders, including major depression.^57^, ^58^ Some works have indicated increased vulnerability of LC neurons to stress and damage.^57^, ^58^, ^59^ Our findings support the notion that LC dysfunction could contribute to the development of depressive symptoms in the early stages of DLB.^59^

Finally, we found significantly higher asymmetry of the LC‐CR between the left and right hemispheres in MCI‐LB patients compared to HCs, with a more pronounced LC degeneration on the right side. In the literature, LC‐CR asymmetry was associated with higher age and worse behavioral performance, such as low emotional memory and regulation, higher anxiety, and depression.^60^ Here we show that the neurodegeneration process may even enhance such asymmetry. Notably, the right‐sided but not left‐sided LC‐CR signal alteration was also described in PD patients compared to HCs,^26^, ^61^ and the LC degeneration was associated with motor symptoms (bradykinesia, motor fluctuations, tremor) and nonmotor symptoms (fatigue, apathy, orthostatic dysregulation, constipation, RBD).^26^, ^43^, ^61^, ^62^ Notably, we did not compare the LC‐CR asymmetry between MCI‐LB and PD patients with or without RBD, and therefore, we cannot comment on the level of LC‐CR asymmetry between potentially more malignant (“body‐first”) and less malignant (“brain‐first”) neuronal Lewy body disease subtypes.

## Conclusion

This study contributes to an in‐depth understanding of the neural underpinnings and specific neurotransmitter involvement of the pre‐dementia stage of DLB. Our findings revealed a significant reduction in the LC integrity of MCI‐LB patients compared to HCs and more involvement of the caudal part of the nucleus in the patient group. This supports a caudal‐rostral direction of pathological α‐synuclein spread. Our results suggest a role for NM‐MRI in identifying early neurodegenerative changes in the LC associated with MCI‐LB. Furthermore, the study highlights the relationship between LC neurodegeneration and cognitive decline. Future research should focus on longitudinal studies to monitor the progression of LC changes over time and investigate the potential of NM‐MRI as a diagnostic or/and prognostic tool for DLB. Further exploration of the relationship between LC neurodegeneration and other nonmotor symptoms in DLB in longitudinal studies may provide valuable insights into the pathophysiology and progression of the disease and guide the development of targeted therapeutic strategies.

## Full financial disclosures of all authors for the previous 12 months

Ž.Ž. received funding from the Ministry of Health of the Czech Republic. L.N. received funding from the Ministry of Health of the Czech Republic, EU Joint Program‐Neurodegenerative Disease, and through project number LX22NPO5107 (MEYS): funded by the European Union—Next Generation EU. L.V. received funding from the Euro‐BioImaging (www.eurobioimaging.eu) ALM and Medical Imaging Node (Brno, CZ). L.B. received funding from the Ministry of Health of the Czech Republic and through project number LX22NPO5107 (MEYS): funded by the European Union—Next Generation EU. K.M. received funding from the Ministry of Health of the Czech Republic and through project number LX22NPO5107 (MEYS): funded by the European Union—Next Generation EU. I.M. received funding from the Ministry of Health of the Czech Republic. I.R. received funding from the Ministry of Health of the Czech Republic, EU Joint Program‐Neurodegenerative Disease, through project number LX22NPO5107 (MEYS): funded by the European Union—Next Generation EU, and by MEYS, LRI CZECRIN (LM2023049).

## Author Roles

(1) Research project: A. Conception, B. Organization, C. Execution; (2) Statistical analysis: A. Design, B. Execution, C. Review and critique; (3) Manuscript: A. Writing of the first draft, B. Review and critique.

Ž.Ž.: 1C, 2B, 3A

L'.N.: 1B, 1C, 2C, 3B

L.V.: 1B, 2C, 3B

L.B.: 1B, 2C, 3B

K.M.: 1C, 2A, 2B, 3B

I.M.: 1C, 2C, 3B

I.R.: 1A, 1B, 2A, 2C, 3B

## Supporting information


**Data S1.** Supporting Information.

## Data Availability

The data that support the findings of this study are available on request from the corresponding author. The data are not publicly available due to privacy or ethical restrictions.
