# Supplementary material for: Early Changes in the Locus Coeruleus in Mild Cognitive Impairment with Lewy Bodies
Source: Mov Disord. 2024 Nov 13;40(2):276–84. doi: 10.1002/mds.30058 (PMC11832806; doi:10.1002/mds.30058)
Supplement: Supplementary file 1 — Data S1. Supporting Information. [file MDS-40-276-s001.docx]

**Supplementary Material**

**Recruitment, inclusion/exclusion criteria**

We recruited participants at risk for prodromal DLB through social media and newspaper ads. These participants were pre-screened by a comprehensive phone interview using a screening questionnaire. Inclusion criteria were: age between 55 and 70 years with subjective cognitive decline, along with at least one of the following symptoms: sleep disturbances (restless sleep, screaming or other noises during sleep, sudden limb movements), bradykinesia and rigidity (slowed movements and muscle stiffness, reduced arm swings, decreased voice loudness and melody of speech), fluctuating memory and attention, visual hallucinations or misperceptions. Additionally, we recorded other symptoms such as reduced olfaction, excessive daytime sleepiness, chronic constipation, symptomatic hypotension (without taking hypertension medication), and mood disorders. These symptoms had to have occurred within the past two months. Exclusion criteria consisted of severe or repeated head injuries, a history of brain inflammation, brain tumors, or intracranial surgery, other known neurological brain diseases such as stroke or epilepsy, major internal diseases, oncological disease, the presence of a pacemaker/defibrillator or MRI-incompatible metal in the body, and disabling musculoskeletal disorders.

**Examination**

In person examinations consist of following scales/tests: Montreal Cognitive Assessment (MoCA) with a cut-off score < 26 or ≤ 26 depending on years of education (Nasreddine et al., 2005), Unified Parkinson’s Disease Rating Scale: Motor Examination (UPDRS III) with a cut-off score > 3 points (Fahn, 1987), Mayo Fluctuation Scale (MFS) with a cut-off score ≥ 3 points (Ferman et al., 2004), Geriatric Depression Scale (GDS) with a cut-off score of 10 points (Yesavage et al., 1982), 5 min of resting-state 256-channel EEG, REM sleep behavior disorder screening questionnaire (RBDSQ) with a cut-off score ≥ 5 points (Stiasny‐Kolster et al., 2007), Neuropsychiatric Inventory (NPI) for detecting hallucinations, misperceptions, or psychosis (Y/N) (Cummings et al., 1994) and Epworth Sleepiness Scale (ESS) with a cut-off score of 11 points (Johns, 1991).

After the screening visit, all participants underwent a detailed neuropsychological cognitive battery and MRI session. The neuropsychological cognitive battery assessed four cognitive domains: memory (brief visuospatial memory test-revised (Benedict, 1997) and the Philadelphia Verbal Learning Test (Bezdicek et al., 2014); attention (Wechsler Adult Intelligence Scale‐III: Letter-Number Sequencing and Digit Symbol Substitution (Wechsler, 1997)); executive functions (semantic and phonemic verbal fluency (Nikolai et al., 2015) and the picture arrangement test (Wechsler, 1997)) and visuospatial functions (Judgment of Line Orientation (Woodard et al., 1998)). The cognitive domain z‐scores were calculated as the average z‐scores of the tests included in the particular domain.

**References:**

Benedict, R.H., 1997. Brief visuospatial memory test--revised. PAR.

Bezdicek, O., Libon, D.J., Stepankova, H., Panenkova, E., Lukavsky, J., Garrett, K.D., Lamar, M., Price, C.C., Kopecek, M., 2014. Development, Validity, and Normative Data Study for the 12-Word Philadelphia Verbal Learning Test [czP(r)VLT-12] Among Older and Very Old Czech Adults. Clin. Neuropsychol. 28, 1162–1181. https://doi.org/10.1080/13854046.2014.952666

Cummings, J.L., Mega, M., Gray, K., Rosenberg-Thompson, S., Carusi, D.A., Gornbein, J., 1994. The Neuropsychiatric Inventory: Comprehensive assessment of psychopathology in dementia. Neurology 44, 2308–2308. https://doi.org/10.1212/WNL.44.12.2308

Fahn, S., 1987. Unified Parkinson’s disease rating scale. Recent Dev. Park. Dis. 153–163.

Ferman, T.J., Smith, G.E., Boeve, B.F., Ivnik, R.J., Petersen, R.C., Knopman, D., Graff-Radford, N., Parisi, J., Dickson, D.W., 2004. DLB fluctuations: Specific features that reliably differentiate DLB from AD and normal aging. Neurology 62, 181–187. https://doi.org/10.1212/WNL.62.2.181

Johns, M.W., 1991. A New Method for Measuring Daytime Sleepiness: The Epworth Sleepiness Scale. Sleep 14, 540–545. https://doi.org/10.1093/sleep/14.6.540

Nasreddine, Z.S., Phillips, N.A., Bédirian, V., Charbonneau, S., Whitehead, V., Collin, I., Cummings, J.L., Chertkow, H., 2005. The Montreal Cognitive Assessment, MoCA: A Brief Screening Tool For Mild Cognitive Impairment. J. Am. Geriatr. Soc. 53, 695–699. https://doi.org/10.1111/j.1532-5415.2005.53221.x

Nikolai, T., Štěpánková, H., Michalec, J., Bezdíček, O., Horáková, K., Marková, H., Růžička, E., Kopeček, M., 2015. Tests of Verbal Fluency, Czech Normative Study in Older Patients. Čes. Slov. Neurol. Neurochir. 78/111, 292–299. <https://doi.org/10.14735/amcsnn2015292>

Stiasny‐Kolster, K., Mayer, G., Schäfer, S., Möller, J.C., Heinzel‐Gutenbrunner, M., Oertel, W.H., 2007. The REM sleep behavior disorder screening questionnaire—A new diagnostic instrument. Mov. Disord. 22, 2386–2393. https://doi.org/10.1002/mds.21740

Wechsler, D., 1997. Wechsler Adult Intelligence Scale--Third Edition. https://doi.org/10.1037/t49755-000

Woodard, J.L., Benedict, R.H.B., Salthouse, T.A., Toth, J.P., Zgaljardic, D.J., Hancock, H.E., 1998. Normative Data for Equivalent, Parallel Forms of the Judgment ofLine Orientation Test. J. Clin. Exp. Neuropsychol. 20, 457–462. https://doi.org/10.1076/jcen.20.4.457.1470

Yesavage, J.A., Brink, T.L., Rose, T.L., Lum, O., Huang, V., Adey, M., Leirer, V.O., 1982. Development and validation of a geriatric depression screening scale: A preliminary report. J. Psychiatr. Res. 17, 37–49. <https://doi.org/10.1016/0022-3956(82)90033-4>

**Evaluation of assumptions of linear regression**

In the models predicting memory z-scores (model A) and combined short-term memory visuospatial memory (model B) from the right LC-CR, the visual assessment of homoscedasticity of residuals (see Figure A and Figure B) revealed two points that deviate significantly from the main cluster indicating potential issues with the model fit. Specifically, two subjects had very low predictor values but high predicted values, standing apart from the rest of the data. After a careful examination of the data quality we decided to exclude these two cases from these particular models as they significantly distort the model parameters.


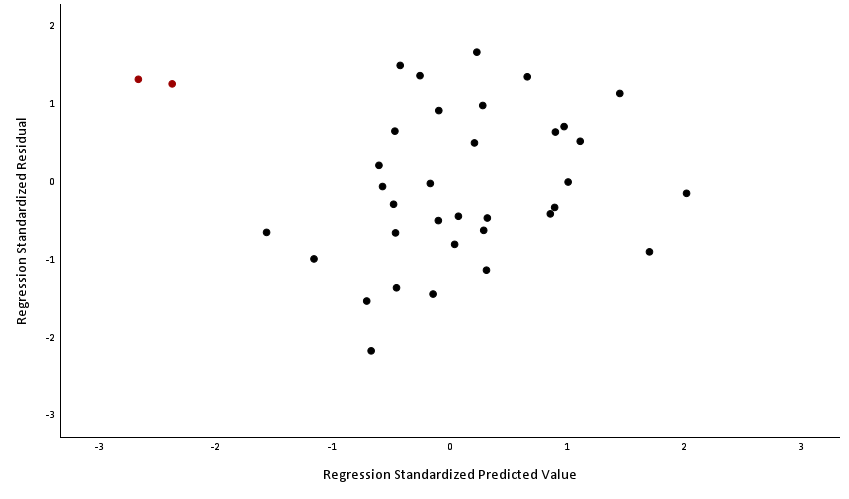


*Figure A depicts two deviating points in red which do not fit the model A.*


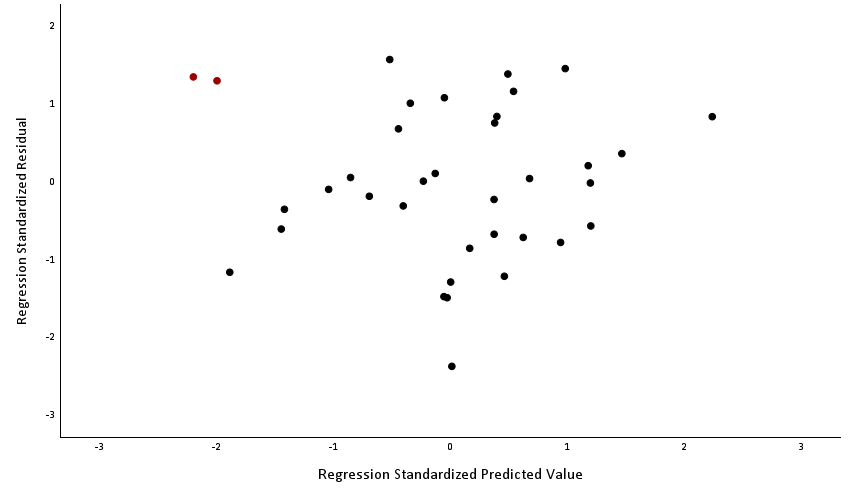


*Figure B depicts two deviating points in red which do not fit the model B.*

| **Dependent variable** | **Predictors** | **Unstandardized B** | **Std. Errors** | **t** | ***p*** |
| --- | --- | --- | --- | --- | --- |
| Memory z-score  (model A) | Constant | -3.165 | 2.236 | -1.415 | .167 |
|  | Right LC | 14.716 | 5.318 | 2.767 | .010 |
|  | Age | .005 | .028 | .190 | .851 |
|  | Education | .036 | .037 | .974 | .338 |
| Visuospatial z-score | Constant | -1.981 | 2.338 | -.847 | .403 |
|  | Right LC | 11.464 | 5.719 | 2.005 | .053 |
|  | Age | -.018 | .030 | -.606 | .549 |
|  | Education | .096 | .050 | 1.921 | .063 |
| BVMT_2 z-score | Constant | -7.035 | 2.979 | -2.362 | .025 |
|  | Right LC | 19.381 | 7.098 | 2.731 | .011 |
|  | Age | .052 | .037 | 1.399 | .172 |
|  | Education | .057 | .052 | 1.110 | .276 |
|  | pTau-181 | -.030 | .046 | -.661 | .514 |
| Visuospatial z-score* | Constant | -1.301 | .618 | -2.106 | .042 |
|  | Right middle LC* | 8.305 | 3.804 | 2.183 | .036 |
| Visuospatial z-score* | Constant | -1.314 | .661 | -1.988 | .055 |
|  | Left middle LC* | 6.518 | 3.176 | 2.052 | .048 |
| Memory z-score*  (model B) | Constant | -2.018 | .559 | -3.608 | .001 |
|  | Right middle LC* | 9.437 | 3.345 | 2.821 | .008 |

*Table 1 Regression coefficients. Note: BVMT – combined score of the short-term memory Brief Visuospatial Memory test; *marks exploratory models in which the dependent variables entered the model as adjusted for the effect of age and education and predictors were adjusted for age. This approach yielded similar results as entering age and education as covariates of interest.*

**Locus Coeruleus**


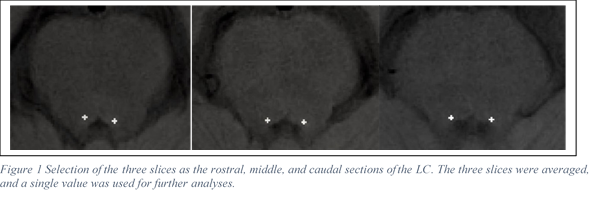


*Figure 1 Selection of the three slices as the rostral, middle, and caudal sections of the LC. The three slices were averaged, and a single value was used for further analyses.*

*Table 2 The results of Mann-Whitney U test of LC-CR between MCI-LB and HC group*

|  | | **W** | | **p** | |
| --- | --- | --- | --- | --- | --- |
| Right LC |  | 1396.0 |  | .021 |  |
| Left LC |  | 1338.0 |  | .055 |  |
| *Note.*  For all tests, the alternative hypothesis specifies that group *HC* is greater than group *MCI-LB*. | | | | | |

*Table 3 Test of Normality (Shapiro-Wilk)*

|  | |  | | **W** | | **p** | |
| --- | --- | --- | --- | --- | --- | --- | --- |
| Right LC |  | HC |  | 0.979 |  | .392 |  |
|  |  | MCI-LB |  | 0.964 |  | .258 |  |
| Left LC |  | HC |  | 0.949 |  | .015 |  |
|  |  | MCI-LB |  | 0.944 |  | .058 |  |
|  | | | | | | | |

*Table 4 Test of Equality of Variances (Levene's)*

|  | | **F** | | **df_1_** | | **df_2_** | | **p** | |
| --- | --- | --- | --- | --- | --- | --- | --- | --- | --- |
| Right LC |  | 1.010 |  | 1 |  | 95 |  | .318 |  |
| Left LC |  | 0.085 |  | 1 |  | 95 |  | .771 |  |

*Table 5 Descriptive statistics of LC-CR values of the MCI-LB and the HC group*

|  | | **Group** | | **N** | | **Mean** | | **SD** | | **SE** | | **Coefficient of variation** | |
| --- | --- | --- | --- | --- | --- | --- | --- | --- | --- | --- | --- | --- | --- |
| Right LC |  | HC |  | 59 |  | 0.144 |  | 0.032 |  | 0.004 |  | 0.224 |  |
|  |  | MCI-LB |  | 38 |  | 0.129 |  | 0.031 |  | 0.005 |  | 0.240 |  |
| Left LC |  | HC |  | 59 |  | 0.194 |  | 0.037 |  | 0.005 |  | 0.189 |  |
|  |  | MCI-LB |  | 38 |  | 0.178 |  | 0.036 |  | 0.006 |  | 0.204 |  |


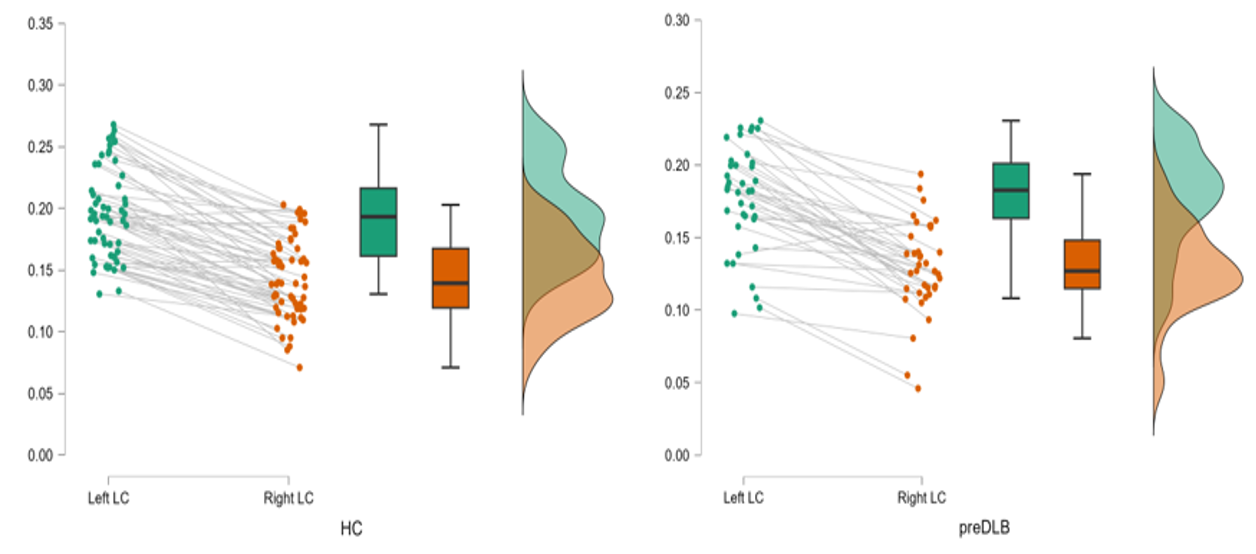


Figure 2 Rainclouds plots showing the difference between the right and the left LC-CNR values between the groups

**Statistics of Brief visuospatial memory test-revised**

*Table 6 Mann-Whitney U-test of BVMT Z scores of the MCI-LB and the HC group*

|  | | **W** | | **df** | | **p** | |
| --- | --- | --- | --- | --- | --- | --- | --- |
| BVMT 2 Z score |  | 1762.500 |  |  |  | < 0.001 |  |
|  | | | | | | | |

*Table 7 Test of Equality of Variances (Levene's)*

|  | | **F** | | **df_1_** | | **df_2_** | | **p** | |
| --- | --- | --- | --- | --- | --- | --- | --- | --- | --- |
| BVMT 2 Z score |  | 4.883 |  | 1 |  | 95 |  | 0.030 |  |

*Table 8 Descriptive statistics of BVMT Z scores of the MCI-LB and the HC group*

|  | | | | | |
| --- | --- | --- | --- | --- | --- |
|  | | **BVMT 2 Z score** | | | |
|  | | **HC** | | **MCI-LB** | |
| Valid |  | 59 |  | 38 |  |
| Mean |  | 0.664 |  | -0.426 |  |
| Std. Deviation |  | 0.796 |  | 1.088 |  |
| Variance |  | 0.634 |  | 1.184 |  |
| Shapiro-Wilk |  | 0.981 |  | 0.981 |  |
| P-value of Shapiro-Wilk |  | 0.502 |  | 0.740 |  |
| Minimum |  | -1.400 |  | -3.000 |  |
| Maximum |  | 2.500 |  | 1.500 |  |


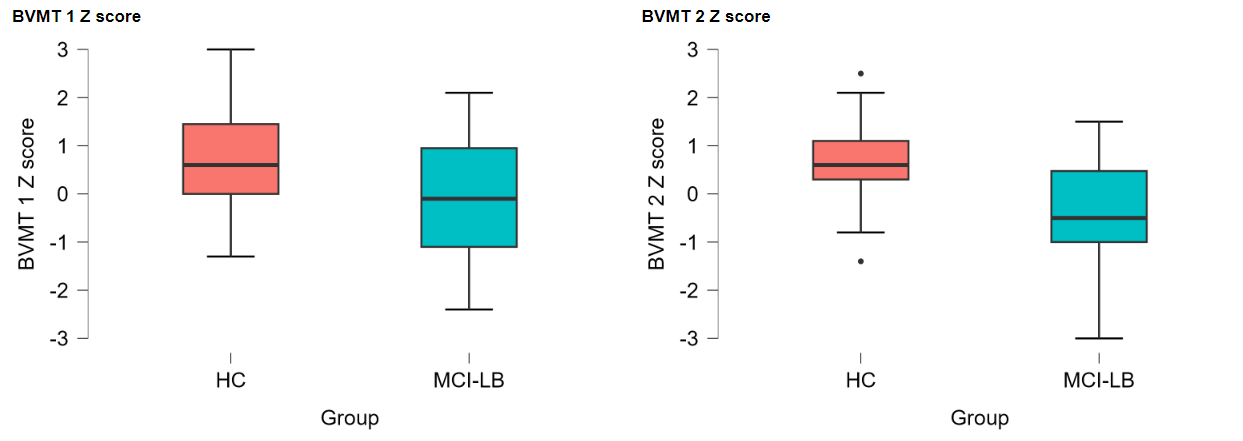


*Figure 3 Boxplots of BVMT Z scores of the MCI-LB and the HC group*

**Exploratory correlation analysis**

*Table 9 Table of correlation between right and left LC-CR and clinical assessment tests*

|  | | | | | | **Pearson** | | | | | **Spearman** | | | |
| --- | --- | --- | --- | --- | --- | --- | --- | --- | --- | --- | --- | --- | --- | --- |
|  | |  | |  | | **r** | | **p** | | | **rho** | | **p** | |
| Right LC |  | - |  | ESS |  | -0.161 |  | 0.166 |  | -0.129 | |  | 0.221 |  |
| Right LC |  | - |  | RBDq |  | -0.104 |  | 0.267 |  | -0.102 | |  | 0.272 |  |
| Right LC |  | - |  | GDS |  | -2.507×10^-4^ |  | 0.499 |  | -0.129 | |  | 0.221 |  |
| Left LC |  | - |  | ESS |  | 0.174 |  | 0.852 |  | 0.180 | |  | 0.860 |  |
| Left LC |  | - |  | RBDq |  | -0.092 |  | 0.291 |  | -0.041 | |  | 0.404 |  |
| Left LC |  | - |  | GDS |  | -0.229 |  | 0.083 |  | **-0.303** | | ***** | **0.032** |  |
| *Note.*  All tests one-tailed, for negative correlation. | | | | | | | | | | | | | | |
|  | | | | | | | | | | | | | | |

**
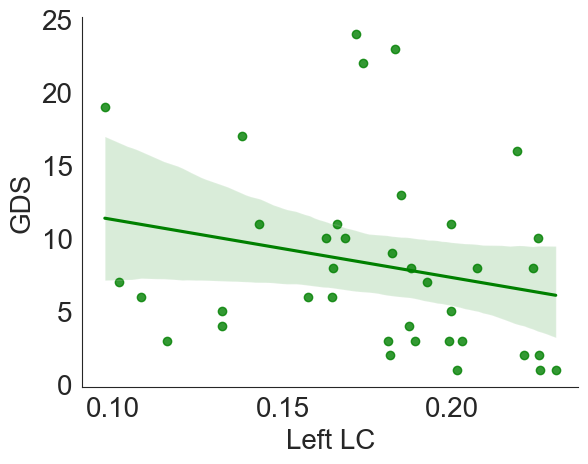
**

*Figure 4 Scatterplot of significant correlation between the left LC-CR and GDS of MCI-LB group*

*Table 10 Mann-Whitney U-test of the segmented LC-CR parts of the MCI-LB and the HC group; (For all tests, the alternative hypothesis specifies that group HC is greater than group MCI-LB)*

|  | | **W** | | **df** | | **p** | |
| --- | --- | --- | --- | --- | --- | --- | --- |
| Right LC Caudal |  | 1553.000 |  |  |  | < 0.001 |  |
| Right LC Middle |  | 1107.000 |  |  |  | 0.543 |  |
| Right LC Rostral |  | 1066.000 |  |  |  | 0.659 |  |
| Left LC Caudal |  | 1560.000 |  |  |  | < 0.001 |  |
| Left LC Middle |  | 1154.000 |  |  |  | 0.405 |  |
| Left LC Rostral |  | 1132.000 |  |  |  | 0.469 |  |
|  | | | | | | | |

*Table 11 Descriptive statistics of the segmented LC-CR parts of the MCI-LB and the HC group*

|  | | **Group** | | **N** | | **Mean** | | **SD** | | **SE** | | **Coefficient of variation** | |
| --- | --- | --- | --- | --- | --- | --- | --- | --- | --- | --- | --- | --- | --- |
| Right LC Caudal |  | HC |  | 59 |  | 0.140 |  | 0.058 |  | 0.008 |  | 0.416 |  |
|  |  | MCI-LB |  | 38 |  | 0.098 |  | 0.049 |  | 0.008 |  | 0.502 |  |
| Right LC Middle |  | HC |  | 59 |  | 0.153 |  | 0.053 |  | 0.007 |  | 0.344 |  |
|  |  | MCI-LB |  | 38 |  | 0.156 |  | 0.043 |  | 0.007 |  | 0.276 |  |
| Right LC Rostral |  | HC |  | 59 |  | 0.138 |  | 0.049 |  | 0.006 |  | 0.353 |  |
|  |  | MCI-LB |  | 38 |  | 0.138 |  | 0.037 |  | 0.006 |  | 0.267 |  |
| Left LC Caudal |  | HC |  | 59 |  | 0.204 |  | 0.061 |  | 0.008 |  | 0.302 |  |
|  |  | MCI-LB |  | 38 |  | 0.157 |  | 0.059 |  | 0.010 |  | 0.379 |  |
| Left LC Middle |  | HC |  | 59 |  | 0.206 |  | 0.057 |  | 0.007 |  | 0.275 |  |
|  |  | MCI-LB |  | 38 |  | 0.202 |  | 0.052 |  | 0.008 |  | 0.256 |  |
| Left LC Rostral |  | HC |  | 59 |  | 0.175 |  | 0.048 |  | 0.006 |  | 0.272 |  |
|  |  | MCI-LB |  | 38 |  | 0.174 |  | 0.051 |  | 0.008 |  | 0.291 |  |
|  | | | | | | | | | | | | | |

**
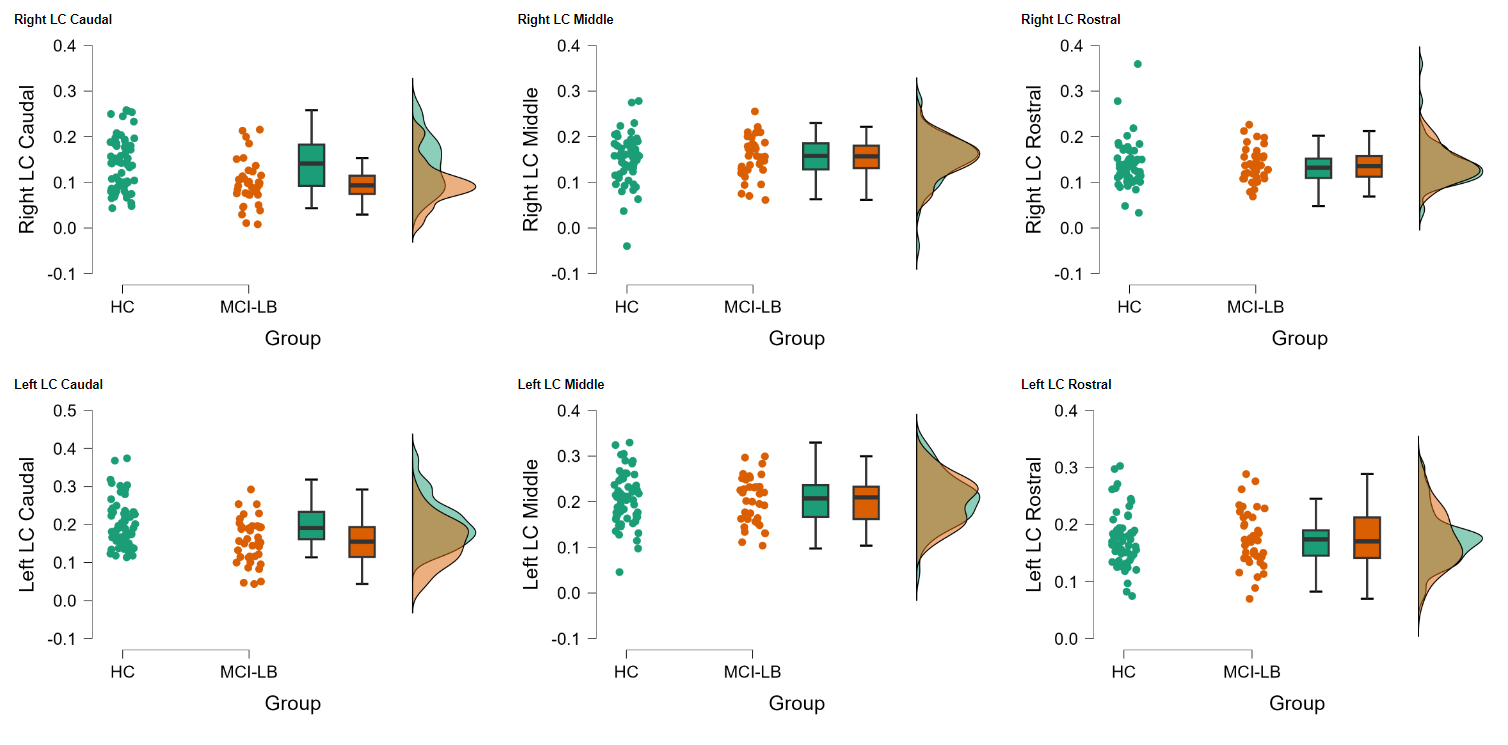
**

*Figure 5 Raincloud plots showing the difference between MCI-LB and HC for the segmented LC-CR*
